# Disparities in access to diagnosis and care in Blantyre, Malawi, identified through enhanced tuberculosis surveillance and spatial analysis

**DOI:** 10.1186/s12916-019-1260-6

**Published:** 2019-01-29

**Authors:** Peter MacPherson, McEwen Khundi, Marriott Nliwasa, Augustine T. Choko, Vincent K. Phiri, Emily L. Webb, Peter J. Dodd, Ted Cohen, Rebecca Harris, Elizabeth L. Corbett

**Affiliations:** 1 Malawi-Liverpool-Wellcome Trust Clinical Research Programme, Queen Elizabeth Central Hospital, Blantyre, Malawi; 2 Department of Clinical Sciences, Liverpool School of Tropical Medicine, Liverpool, UK; 3 Helse-Nord TB Programme, College of Medicine, University of Malawi, Blantyre, Malawi; 4 Department of Infectious Disease Epidemiology, London School of Hygiene and Tropical Medicine, London, UK; 5 MRC Tropical Epidemiology Group, London School of Hygiene and Tropical Medicine, London, UK; 6 School of Health and Related Research, University of Sheffield, Sheffield, UK; 7 Yale School of Public Health, Yale University, New Haven, USA; 8 Department of Clinical Research, London School of Hygiene and Tropical Medicine, London, UK

**Keywords:** Tuberculosis, HIV, Epidemiology, Surveillance, Spatial analysis, Bayesian regression analysis, Inequality, Access to care, Poverty, Gender

## Abstract

**Background:**

A sizeable fraction of tuberculosis (TB) cases go undiagnosed. By analysing data from enhanced demographic, microbiological and geospatial surveillance of TB registrations, we aimed to identify modifiable predictors of inequitable access to diagnosis and care.

**Methods:**

Governmental community health workers (CHW) enumerated all households in 315 catchment areas during October–December 2015. From January 2015, government TB Officers routinely implemented enhanced TB surveillance at all public and private TB treatment registration centres within Blantyre (18 clinics in total). This included collection from registering TB patients of demographic and clinical characteristics, a single sputum sample for TB microscopy and culture, and geolocation of place of residence using an electronic satellite map application. We estimated catchment area annual TB case notification rates (CNRs), stratified by microbiological status. To identify population and area-level factors predictive of CHW catchment area TB case notification rates, we constructed Bayesian spatially autocorrelated regression models with Poisson response distributions. Worldpop data were used to estimate poverty.

**Results:**

In total, the 315 CHW catchment areas comprised 753,489 people (range 162 to 13,066 people/catchment area). Between 2015 and 2017, 6077 TB cases (61% male; 99% HIV tested; 67% HIV positive; 55% culture confirmed) were geolocated, with 3723 (61%) resident within a CHW catchment area. In adjusted models, greater distance to the nearest TB registration clinic was negatively correlated with TB CNRs, which halved for every 3.2-fold (95% CI 2.24–5.21) increase in distance. Poverty, which increased with distance from clinics, was negatively correlated with TB CNRs; a 23% increase (95% CI 17–34%) in the mean percentage of the population living on less than US$2 per day corresponded to a halving of the TB case notification rates.

**Conclusions:**

Using enhanced surveillance of TB cases in Blantyre, we show an ecological relationship consistent with an ‘inverse care law’ whereby poorer neighbourhoods and those furthest from TB clinics have lower relative CNRs. If confirmed as low case detection, then pro-poor strategies to facilitate equitable access to TB diagnosis and treatment are required.

**Electronic supplementary material:**

The online version of this article (10.1186/s12916-019-1260-6) contains supplementary material, which is available to authorized users.

## Background

A strong socio-economic gradient between poverty and incidence of tuberculosis (TB) has been demonstrated at both population and individual levels [1, 2], with the poorest in society having the greatest risk of developing active TB disease [3]. Poor housing quality, malnutrition, HIV and unhealthy behaviours such as smoking appear to be the major intermediary determinants [4]. People living in poverty also face the greatest difficulties in accessing TB diagnosis and treatment [5] and frequently experience catastrophic care-seeking costs [6]. The World Health Organization (WHO) End-TB strategy has called for ambitious policies to ensure all TB cases are detected and treated and to ensure that no person affected by TB faces socio-economic ruin [7]. However, in sub-Saharan Africa, a substantial ‘detection gap’ exists, with an estimated 51% of prevalent TB cases remaining undiagnosed in the community in 2016 [1].

There is likely substantial heterogeneity in the determinants of incident active TB and in access to TB diagnosis care within cities [8], meaning that preventive interventions and improved access to high-quality TB services targeted towards local neighbourhoods with the greatest need could plausibly result in improvements in TB control [9]. However, identifying these ‘hot-spots’ of inequitable access to TB care depends on integrating high-quality epidemiological and microbiological surveillance with geospatial technologies. Lower case notification rates from certain areas may reflect either worse access to care or lower TB burden. Where lower notification rates coincide with higher levels of known risk factors for TB (e.g. poverty), they may instead reflect barriers to access rather than a relative absence of TB.

We report on the implementation and evaluation of an enhanced TB surveillance programme in Blantyre, Malawi, where nearly one-in-five adults are HIV positive [10]. We aimed to use high-quality spatially resolved surveillance data to support public health practitioners and policymakers to identify the most important and modifiable barriers to TB diagnosis and treatment. We hypothesised that neighbourhood characteristics such as poverty rates, age and sex structure, and distance from TB registration clinics might be correlated with TB case notification rates, and so guide the implementation of interventions to improve access to TB diagnosis and prevention.

## Methods

### Study site and population

Blantyre is a major urban centre located in the south of Malawi. Blantyre is divided into non-overlapping geographical areas, each served by locally resident community health workers (CHW) known as Health Surveillance Assistants. CWHs are employed by the Ministry of Health of Malawi and serve as a link between health facilities and communities.

### Enumeration of Health Surveillance Assistant areas

To estimate population denominators for the study, we defined boundaries around 315 CHW catchment areas, excluding business and industrial areas and the most affluent areas of the city. Through community engagement activities, and with the support of the Blantyre District Health Officer, we provided training in census enumeration to CHWs in Blantyre. Between 10 October 2015 and 30 December 2015, CHWs, accompanied by study Research Assistants, undertook a circumferential walk around their catchment area to record sets of boundary coordinates using global positioning satellite devices. Where there was overlap between two contiguous CHW catchment areas, the circumferential walks were repeated and boundaries revised to ensure that no areas overlapped.

CHWs and Research Assistants subsequently undertook a door-to-door census from all dwellings within each CHW catchment area. We defined a dwelling to be a physical structure within which members of one or more households had slept in the previous week. We excluded dwellings that were derelict or abandoned. We defined a household to be a person living alone or a group of people living together who shared meals together and who may have been related or unrelated. The head of each household (or, in their absence, the next most senior household member) was interviewed using a structured census questionnaire that recorded the number of households and people residing within the dwelling. Five per cent of enumerated households were randomly selected, revisited and re-enumerated by a separate Research Assistant team for quality control purposes.

### Enhanced surveillance of tuberculosis case notifications

Within Blantyre’s administrative border, there are a total of 18 health facilities where TB Officers employed by the Ministry of Health register TB cases and initiate treatment for TB. As TB diagnostic and treatment centres are not evenly spread through Blantyre, geographical proximity to diagnostic services may be an important predictor of accessing care.

At all health facilities in the city, patients are investigated for TB by health workers, usually with an initial symptom screen and subsequent examination of a diagnostic sample, most commonly sputum smear microscopy. Once a decision to initiate TB treatment has been made by a health worker—either on the basis of a positive microbiological sample or on clinical decision—the patient is referred to their nearest TB registration clinic, which will usually be located within the same health facility. Here, Ministry of Health TB Officers initiate TB treatment and register the patient using the National TB Register, recording demographic and clinical characteristics. In this study, we implemented enhanced TB surveillance at the point of TB registration.

We identified all TB Officers who registered TB cases at the 18 health facilities in Blantyre (one public and two private hospitals, 13 public and one private primary health care centres, and one prison clinic), and provided study-specific training in enhanced TB surveillance activities. In addition to registering TB cases using the National TB Programme register, TB Officers were asked to complete an enhanced surveillance form that recorded registration centre, age, sex, any sputum smear results taken as part of routine care (positive, negative or not done), TB treatment category (using standard Ministry of Health/WHO categories), TB classification (pulmonary or extrapulmonary TB) and HIV status (positive, negative or unknown). Monthly, study coordinators held joint meetings with all TB Officers and research team members where clinic TB Registers were reconciled with enhanced surveillance data.

### Geolocation of TB cases

Following registration of a TB case, TB Officers used a satellite map application (electronic patient locator [ePAL]) that we had previously developed and evaluated to geolocate the physical location of each case’s dwelling. ePAL and its precursor—the Mapbook—have been described before [11, 12]. In brief, we purchased high-resolution satellite maps from the European Space Agency, and CHWs geotagged important points of reference within their catchment areas, including schools, clinics, bars, roads, churches and other physical landmarks. Throughout the study period, points of reference were periodically added-to and updated. Within the ePAL application map screen, TB patients searched for points of interest known to be near their dwelling using a text matching algorithm, then scrolled and zoomed to identify their dwelling; a subsequent long-press on the map screen recorded a set of coordinates from the selected dwelling location.

### Microbiological surveillance

TB Officers asked all registering adult TB cases (regardless of TB category or classification) to submit a single sputum sample for smear and culture. Sputum samples were collected daily from each registration centre and transported in cooler boxes to the TB Research Laboratory at the College of Medicine, University of Malawi, for fluorescence microscopy and MGIT culture. Patients with positive results requiring additional clinical input were traced and linked to care.

### Statistical analysis

We took the total population denominators of each CHW catchment area from the study census enumeration. CHW catchment area population densities were calculated and plotted in people per square kilometre. Using the ‘st_distance’ function in the R ‘sf’ package [13], we estimated the shortest direct Cartesian distance between the centroid of each CHW catchment area and the nearest TB registration clinic, without accounting for interceding physical structures. We overlaid 1-km gridded raster images containing data on the percentage of the population living on less than US $2 per day obtained from the Worldpop Project [14] onto CHW catchment area boundaries; where CHW catchment areas were covered by more than one 1-km grid square, we took the mean of the percentages.

We used the ‘st_within’ function in the R ‘sf’ package [13] to classify the CHW catchment area of residence of each case. Where notified TB cases were resident within a study-mapped CWH catchment area, we classified them as CHW catchment area residents; where TB cases were resident in an area of the city not mapped by study activities, or in another part of the country, we classified them as non-CHW catchment area resident. We compared the characteristics of TB cases that resided within and outside of study CHW catchment areas using proportions and means. Using census denominators, we estimated CHW catchment area TB case notification rates (CNRs) per 100,000 per year and disaggregated by TB microbiological status (smear positive or culture positive for *Mycobacterium tuberculosis* on study enhanced surveillance sample vs. all TB cases).

Using the CHW catchment area as the unit of analysis, we undertook two Bayesian conditional autocorrelated regression modelling analyses with Poisson response distributions [15]: the first to estimate all TB CNRs and the second to estimate TB CNRs that were microbiologically confirmed by the study laboratory as part of enhanced surveillance activities. We included within both models linear predictors for the log_10_(Poisson rate), including log_10_ population density, mean number of people per household, log_10_ Cartesian distance in metres from the nearest TB registration centre, percentage of population living on less than US $2 per day, adult male-to-female ratio, the percentage of the population that was aged 15 years or older and—as a marker of late presentation for diagnosis—the ratio of sputum smear-positive to smear-negative notified cases (on smears done by the routine health system; where it was not possible to estimate a ratio, either because no cases were notified from that CHW catchment area or there were no smear-positive cases, we inputted a value of 1). We additionally included an offset term for log_10_ population size of each CHW catchment area so as to model per capita rates. We included a diffuse Gaussian prior with mean 0 and standard deviation 10 on each covariate and the population-averaged intercept.

The spatial conditional autoregressive structure [16] used to account for spatial correlations in TB CNRs was a Besag-York-Mollié prior using a nearest-neighbours matrix method where the two catchment areas with the shortest Cartesian distance between CHW centroids were classified as neighbours. To determine the optimal nearest-neighbours correlation matrix, we plotted network graphs of CHW catchment areas, varying from *k* = 0 (no spatial autocorrelation prior) to *k* = 6 (each CHW catchment area was correlated with five other CHW catchment areas). To select the final models, we constructed for each analysis seven Bayesian regression models including priors *k =* 0 to *k =* 6, compared their widely applicable information criteria (WAIC) statistics [17] and selected the model with the lowest WAIC value.

Samples from posterior distributions were drawn using Markov chain Monte Carlo (MCMC) methods, specifically the non-U-turn sampler (NUTS) compiled in Stan using the ‘brms’ version 2.4.0 package [18] running in R version 3.5.1 (R Foundation for Statistical Computing, Vienna). For both analyses (all TB CNRs and microbiologically confirmed TB CNRs), we ran three chains of 4000 iterations with 1000 warm-up samples. We checked model convergence by estimating Gelman-Rubin statistics, inspecting trace plots, plotting residuals and plotting the geographical distribution of model-fitted to observed case notifications. We estimated posterior means and 95% credible intervals and plotted sample posterior distributions to examine the strength of evidence for covariate correlation with TB CNRs per CHW catchment area.

## Results

### Population characteristics

**Fig. 1:**
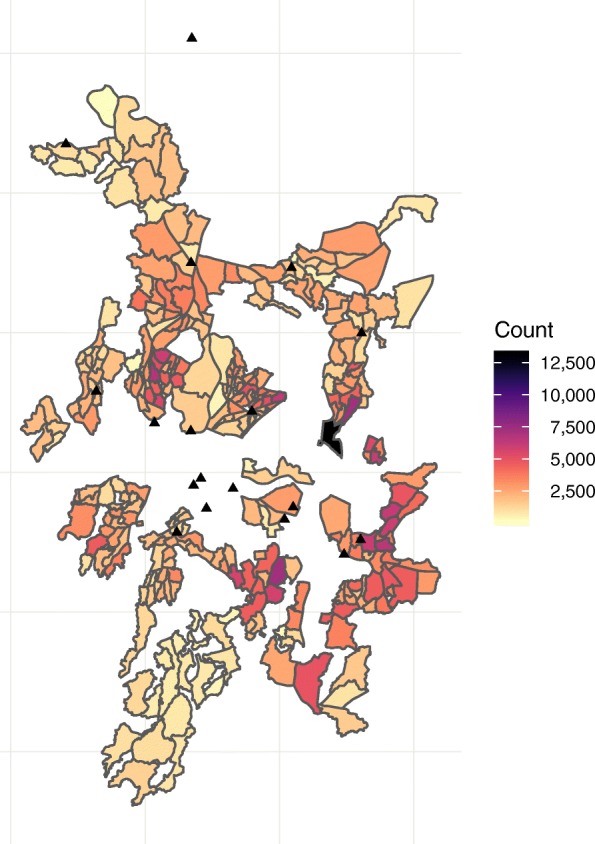
Health Surveillance Assistant boundaries and populations 2015, Blantyre, Malawi. Population counts from study census conducted from October to December 2015. Boundaries are community health worker catchment areas, recorded by circumferential walks recording global positioning satellite coordinates by community health worker study team during October to December 2015. Black triangles are TB registration clinics. White areas in the centre of the map are mountainous areas or business districts with few residents that were not enumerated in the census or mapped by CHWs. The black triangle in the far north of map is a health centre with a TB registration clinic located outside of Blantyre District that may be used by Blantyre residents; to increase accuracy of case notification rates, we captured TB registrations at this clinic

A total of 753,489 people resident within 315 CHW catchment areas were enumerated (Fig. 1). CHW populations ranged from 162 people to 13,066 people, with a mean of 2392 (standard deviation 1504) and median of 2043 (25th percentile 1338; 75th percentile 3035). The mean catchment area male-to-female adult ratio was 1.04 and ranged from 0.83 to 1.35 (Additional file 1: Figure S1). Overall, 61.5% of catchment area residents were aged 15 years or older, with cluster level percentages of adults ranging from 44.4 to 78.0%. The mean percentage of the population living in poverty (< $US2 per day) was 32% (range 13 to 79%).

### TB case notifications

Between January 2015 and December 2017, 7799 TB cases were registered for TB treatment at the 18 TB registration clinics. Overall, 6077 (78%) registering TB cases were captured by the ePAL geolocation system. Of these, 3723 cases were resident within a CHW catchment area and 2354 were not: 1722 residing in a District outside Blantyre city, and 632 resided inside Blantyre city but outside of a study CHW catchment area or in an area of the city that had not been mapped by study activities and where enhanced surveillance GPS coordinates could not be obtained.

Characteristics of CHW-resident and non-CHW-resident TB cases were broadly similar, but with some important differences (Table 1). Overall, 61% (3727/6077) of TB cases were male, 60% (3629/6076) had pulmonary TB and 60% (1536/4089) were recorded as being sputum smear positive in the clinic TB register. Ascertainment of HIV status by TB Officers was very high, with HIV status recorded in > 99% of cases. Overall, 67% (3964/5915) of TB cases were HIV positive. However, compared to non-CHW area-resident cases, CWH area-resident cases were more likely to be male (64% vs. 58%), have pulmonary TB (62% vs. 55%), be sputum smear positive on routine clinic sample (62% vs. 56%) and on study laboratory sample (45% vs. 39%) and be culture positive on study laboratory sample (59% vs. 49%).

**Table 1:** Baseline characteristics of TB cases recorded in enhanced surveillance system in Blantyre

	CHW resident (*N* = 3723)	Non-CHW resident (*N* = 2354)	Total (*N* = 6077)
Year			
2015	994 (26.7%)	666 (28.3%)	1660 (27.3%)
2016	1314 (35.3%)	886 (37.6%)	2200 (36.2%)
2017	1415 (38.0%)	802 (34.1%)	2217 (36.5%)
Sex			
Female	1358 (36.5%)	992 (42.1%)	2350 (38.7%)
Male	2365 (63.5%)	1362 (57.9%)	3727 (61.3%)
Age (mean, sd)	35.0 (13.5)	36.1 (16.0)	35.4 (14.5)
TB classification			
Missing	1	0	1
Extrapulmonary TB	1395 (37.5%)	1052 (44.7%)	2447 (40.3%)
Pulmonary TB	2327 (62.5%)	1302 (55.3%)	3629 (59.7%)
Clinic registration sputum smear status			
Missing/not done	2087	1437	3524
Smear negative	618 (37.8%)	399 (43.5%)	1017 (39.8%)
Smear positive	1018 (62.2%)	518 (56.5%)	1536 (60.2%)
HIV status			
Missing	99	63	162
HIV negative	1146 (31.6%)	805 (35.1%)	1951 (33.0%)
HIV positive	2478 (68.4%)	1486 (64.9%)	3964 (67.0%)
Laboratory sputum smear status			
Not done/lab issue	911	698	1609
Scanty positive	18 (0.6%)	5 (0.3%)	23 (0.5%)
Smear negative	1524 (54.2%)	1000 (60.4%)	2524 (56.5%)
Smear positive	1270 (45.2%)	651 (39.3%)	1921 (43.0%)
Laboratory sputum TB culture status			
Not done/lab issue	1013	758	1771
Culture negative	1113 (41.1%)	808 (50.6%)	1921 (44.6%)
Culture positive	1597 (58.9%)	788 (49.4%)	2385 (55.4%)
Laboratory TB species			
Not done/lab issue	2230	1625	3855
*Mycobacterium tuberculosis*	1493 (100.0%)	729 (100.0%)	2222 (100.0%)
Microbiologically confirmed TB			
Microbiologically confirmed TB	1700 (45.7%)	845 (35.9%)	2545 (41.9%)
Not microbiologically confirmed TB	2023 (54.3%)	1509 (64.1%)	3532 (58.1%)

*CHW* community health worker

Sputum samples taken for TB culture as part of enhanced TB surveillance activities were successfully collected and reported for 71% (4306/6077) of cases overall and 73% (2170/3723) of CHW catchment area-resident cases. Overall, 59% of TB cultures done among CHW catchment area-resident cases were positive, with all speciated as *Mycobacterium tuberculosis.* A further 103 cases were sputum smear positive, but culture negative, meaning that for this analysis, 54% (1700/3723) of CHW catchment area-resident cases were classified as microbiologically confirmed.

### TB case notification rates

**Fig. 2:**
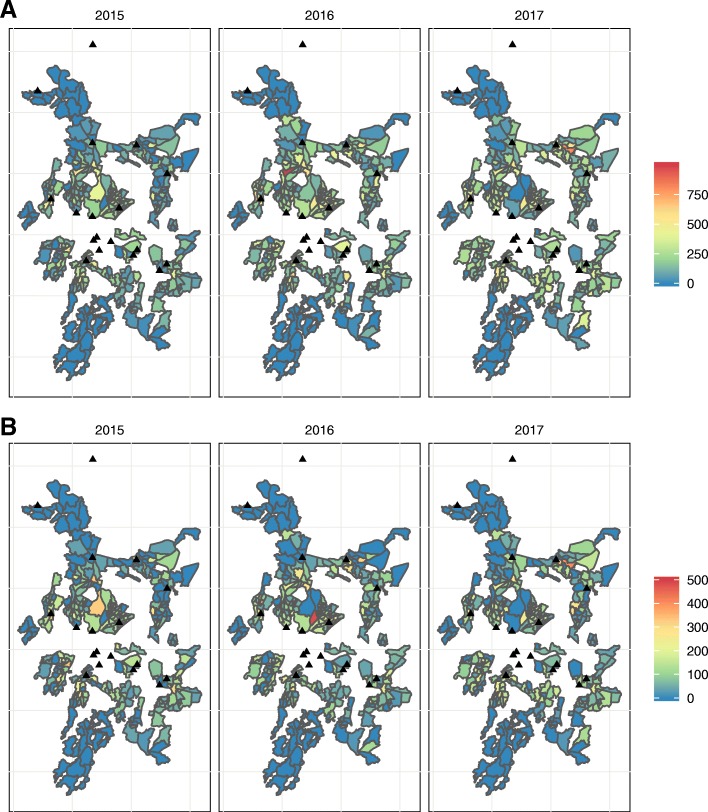
Annual Health Surveillance Assistant TB case notification rates, Blantyre, Malawi: 2015–2017. **a** All TB cases. **b** Microbiologically confirmed TB cases. Case notification rates per 100,000 population per year. Boundaries are community health worker catchment areas. Black triangles are TB registration clinics. White areas in the centre of the map are mountainous areas or business districts with few residents that were not enumerated in the census. The black triangle in the far north of the map is a health centre with a TB registration clinic located outside of Blantyre District that may be used by Blantyre residents; to increase accuracy of case notification rates, we captured TB registrations at this clinic

Annual CHW catchment area TB CNRs ranged from 0 to 986 per 100,000 population per year. Across all 3 years, CNRs were highest in the densely populated areas of central urban Blantyre (Fig. 2). Catchment areas in the poorer and less densely populated outer suburbs recorded very few TB case notifications.

### Factors associated with TB case notifications

We ran seven Bayesian regression analyses to determine the optimal spatial autocorrelation prior for inclusion in each final regression analysis (all TB CNRs and microbiologically confirmed TB CNRs)—Additional file 2: Figure S2 and Additional file 3: Figure S3. For analysis 1 (all TB cases) and analysis 2 (microbiologically confirmed TB cases), a *k* = 6 and *k* = 4 nearest-neighbour correlation prior respectively provided the lowest WAIC statistic, and there was evidence for better fit when compared to models without spatial autocorrelation priors (Additional file 4: Figure S4).

**Fig. 3:**
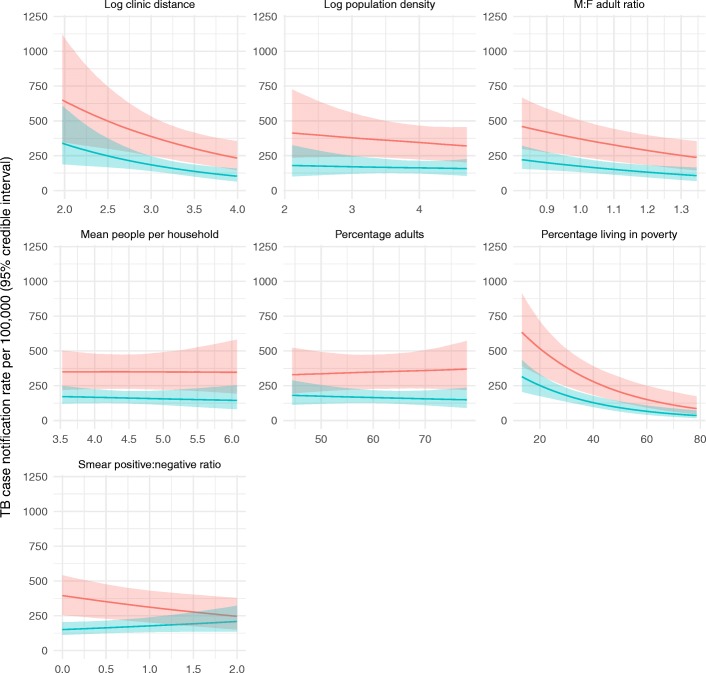
Marginal effects of covariates on TB case notification rates, Blantyre, 2015–2017. Red: analysis 1 (all TB); blue: analysis 2 (microbiologically confirmed TB). Estimated by fitting a Bayesian spatial regression model with Poisson response, a *k* nearest-neighbours conditional spatial autocorrelation prior (with *k* = 6 for analysis 1 and *k* = 4 for analysis 2), with linear terms fitted for community health worker catchment area log_10_ population density, adult M:F ratio, mean number of people per household, log_10_ Cartesian distance from geographical centroid to the nearest TB clinic, percentage of population aged 15 years or older, mean percentage living on less than US $2 per day, offset term for log_10_ population size, and with weakly informative prior on the population-level effects intercept (Gaussian: mean = 0, sd = 10), and predictor intercept (Gaussian, mean = 0, sd = 10). Lines are medians, estimated from 100 draws from posterior samples. Shaded areas are 95% credible intervals. Each marginal distribution estimated by holding all other variables constant at their median

Both final regression analyses (analysis 1: all TB case notification rates; analysis 2: microbiologically confirmed TB case notification rates) showed evidence of convergence with stable mixing across chains. Gelman-Rubin statistics were 1.00 for all covariates, and residual plots showed evidence of good model fit (Additional file 4: Figure S4). Posterior probability distributions for fully adjusted covariate effects are shown in Table 2, and marginal effect plots are shown in Fig. 3.

**Table 2:** Posterior distributions of population-level covariates in a Bayesian spatial model for predicting tuberculosis case notification rates in Blantyre, Malawi: 2015–2017

	Adjusted relative rate	Lower 95% credible interval	Upper 95% credible interval
Analysis 1: all TB cases			
Mean number of people per household	1.00	0.78	1.27
Log_10_ population density (people/km^2^)	0.90	0.70	1.16
Log_10_ distance to nearest TB clinic (m)	0.60	0.42	0.86
Mean proportion of population living in poverty	0.97	0.96	0.98
Adult male-to-female ratio	0.28	0.11	0.72
Proportion of population aged ≥15 years	1.00	0.98	1.02
Sputum smear positive to negative ratio	0.79	0.65	0.97
Analysis 2: microbiologically confirmed TB cases			
Mean number of people per household	0.93	0.70	1.24
Log_10_ population density (people/km^2^)	0.95	0.70	1.29
Log_10_ distance to nearest TB clinic (m)	0.55	0.36	0.84
Mean proportion of population living in poverty	0.97	0.95	0.98
Adult male-to-female ratio	0.24	0.08	0.74
Proportion of population aged ≥ 15 years	0.99	0.97	1.02
Sputum smear positive to negative ratio	1.18	0.95	1.48

Estimated by fitting a Bayesian spatial regression model with Poisson response, a *k* nearest-neighbours conditional spatial autocorrelation prior (with *k* = 6 for analysis 1 and *k* = 4 for analysis 2), with linear terms fitted for community health worker catchment area log_10_ population density, adult M:F ratio, mean number of people per household, log_10_ Cartesian distance from geographical centroid to the nearest TB clinic, percentage of population aged 15 years or older, mean percentage living on less than US $2 per day, offset term for log_10_ population size, and with weakly informative prior on the population-level effects intercept (Gaussian: mean = 0, sd = 10), and predictor intercept (Gaussian, mean = 0, sd = 10)

Adjusting for the effects of other covariates, we found strong evidence for a negative correlation between CHW catchment area TB CNRs and greater Cartesian distance between the catchment area centroid and the nearest TB clinic (analysis 1: relative rate [RR] per log_10_ increase in clinic distance 0.60, 95% credible interval [CI] 0.42–0.86); analysis 2: RR 0.55, 95% CI 0.36–0.84). For analyses 1 and 2, the TB case notification rate halved for every 3.2-fold (95% CI 2.24–5.21) and 3.5-fold (95% CI 2.28–6.86) increase in distance respectively.

We additionally found strong evidence for a negative correlation between TB CNRs and the percentage of CHW catchment area population that were living in poverty (analysis 1: RR 0.97, 95% CI 0.96–0.98; analysis 2: RR 0.97, 95% CI 0.95–0.98). In adjusted analysis, for analysis 1, a 23% (95% CI 17–34%) increase in the mean percentage of the population living on less than US$2 per day corresponded to a halving of the TB CNRs. The effect was similar for analysis 2 (23%, 95% CI 14–34%).

For both analyses, there was evidence in support of a correlation between higher TB CNRs and lower CHW catchment area male-to-female adult ratios (i.e. where a greater percentage of the adult population was female). For analysis 1, a 1.8% (95% CI 1.03–7.07%) decrease in the male-to-female adult ratio was associated with a 10% reduction in the CNR for all TB cases. For analysis 2 (microbiologically confirmed cases), each 1.6% (95% CI 0.91–7.72%) decrease in the male-to-female adult ratio was associated with a 10% reduction in the CNR.

The sputum smear positive to negative ratio (from samples collected for TB diagnosis by the routine health system) showed mixed results. For analysis 1 (all TB cases), the TB CNR halved for every 2.9% (95% CI 1.61–22.76%) decrease in the smear positive to negative ratio. In contrast, for analysis 2 (microbiologically confirmed TB), there was little evidence of an association between TB CNRs and the smear positive to negative ratio (RR 1.19, 95% CI 0.94–1.47).

There was little evidence to support correlations between TB CNRs and population density, mean number of people per household or the proportion of the population aged 15 years or older.

## Discussion

Through enhanced TB surveillance in Blantyre, Malawi, we found evidence supporting the ‘inverse case law’, with adjusted TB case notification rate ratios for the poorest neighbourhoods, and those furthest from TB registration clinics, being substantially lower than for less poor neighbourhoods and for those in closer proximity to TB diagnosis and care centres. Evidence was consistently strong in models investigating microbiologically confirmed TB disease and all TB cases, supporting our hypothesis that poor access to TB diagnosis and treatment is predominately determined by poverty, and disproportionately affects the most disadvantaged in society. If a high burden of undiagnosed TB is confirmed through future prevalence surveys, then policymakers should strongly consider prioritising the implementation of pro-poor interventions to improve access to TB services in these underserved neighbourhoods. This could include expanding or relocating primary health care centres with comprehensive TB and HIV services [19], periodic mobile TB screening camps [20] or targeted community-wide active case-finding interventions [21, 22]. There may also be a potential impact from wider development interventions, not specific to TB.

Cities—and in particular informal settlements—provide conditions highly conducive to the spread of infectious disease [23], although it is clear that there is considerable social and geographical heterogeneity in the prevalence and incidence of infections, including TB [2, 9]. The prevalence of undiagnosed active TB is known to be high in many urban Africa cities [21, 22]. However, understanding of how and where to best implement TB control strategies in resource-limited settings has been hampered by a lack of census data and TB case notification data disaggregated by age, sex and HIV status and at lower-level geospatial boundaries.

In Blantyre, we successfully implemented enhanced TB surveillance in an extremely densely populated setting, including routine sputum sample collection for mycobacterial culture and geolocation using an innovative electronic application. We believe this approach could be widely applied in other similar settings and would be beneficial to public health authorities, providing neighbourhood-level data on underserved areas to inform TB care and prevention strategies. Critical to achieving the high levels of case-capture were the partnerships we established with key stakeholders, including the Malawi National TB Programme and Blantyre District Health Office, and with the Blantyre TB Officers and community health workers who undertook most of the enhanced surveillance activities.

We additionally found strong evidence that neighbourhoods with lower male-to-female adult ratios (i.e. a higher proportion of women) had higher TB CNRs even after adjusting for other important determinants of access to care. Although we cannot speculate about the causal nature of the relationship between neighbourhood sex ratios and TB case notification rates, if confirmed by prevalence survey data, this suggests that lower case notification rates may, in part, be driven by male barrier to TB diagnosis. Community TB prevalence and case notifications are known to be substantially higher for men than women [24], perhaps driven by gendered patterns of social mixing and barriers in care-seeking [25]. Areas in Blantyre with the highest male-to-female ratios are centred around extremely densely populated trading areas, where young men migrate for work. For such individuals, intense economic pressure may mean that seeking care for symptoms of TB is not a priority, a hypothesis that is supported by our previous qualitative work with symptomatic men in the city [25]. Conversely, residents of neighbourhoods where sex ratios were equal, or where women outnumber men, may be relatively more stable, with social stability a prerequisite for prioritising individual or household health.

As a further potential marker of poor access and late presentation for care that could be investigated using routine programmatic surveillance data, we investigated associations between sputum smear positive to negative notification ratios (on samples collected by the routine TB programme) and neighbourhood TB case notification rates. Smear positivity is a marker of infectivity that increases with diagnostic delay but, once present, is considerably easier for routine programmes to diagnose than smear-negative TB; thus, greater diagnostic access barriers are likely to be reflected by a higher proportion of smear-positive patients. Here, we show that TB case notification rates halved with every 2.4-fold increase in the sputum smear positive to negative ratio. This supports our hypothesis that late and/or under-diagnosis of TB from the poorest, more distant neighbourhoods was explained by poor access to diagnosis rather than by less prevalent disease. However, confirming this would need neighbourhood-level data from TB prevalence surveys (and in particular prevalence-to-notification ratios), ideally combined with local data on other intermediary determinants.

There were a number of strengths and limitations to this study. Overall, we successfully recorded clinical and demographic data from 78% (6077/7799) of all registering TB cases at all 18 health facilities in urban Blantyre. We additionally completed microbiological surveillance by sputum culture from 71% of captured cases, and geolocated 70% of all TB cases registered at city health centres. We concentrated mapping activities on the largest and most deprived neighbourhoods of the city and excluded business districts and the most affluent neighbourhoods. Thus, we will have systematic under-sampled TB patients from middle-class homes. Reflecting these different underlying populations, non-CHW catchment area-resident cases did have different characteristics compared to CHW-resident cases. Additionally, because we could only collect geospatial data from TB cases who were resident within neighbourhoods mapped by our study activities, we are unable to include TB cases resident outwith mapped areas in our regression analysis. There therefore may be important predictors of access to TB diagnosis and care among populations excluded from the present analysis that we were unable to identify. Surveillance data were collected by routine Ministry of Health employees (TB Officers) using the electronic GPS application and not by research staff. Being programmatic data, we anticipate some imprecision in geolocating TB patients. The household enumeration data was limited to basic data on age and sex, and so we are unable to estimate correlations with important intermediary determinants—notably HIV prevalence; the relationship with sputum smear positivity rates at the population level remains uncertain without detailed HIV prevalence data. We relied on data from the Worldpop Project from for estimating neighbourhood-level poverty. TB cultures were not done on 27% of CHW-resident TB patients who did not submit sputum, including 17% of pulmonary TB cases: this is likely to include a bias against less gravely ill patients too sick to produce sputum, or started on TB treatment out-of-hours while inpatients. Overall, 58% of notified TB cases initiated on TB treatment by the National TB Programme in study health facilities were microbiologically confirmed by the study laboratory, reflecting the programmatic and diagnostic challenges in confirming TB disease in low-resource settings. Finally, without supporting epidemiological data, our hypothesis relating to under-diagnosis in the more remote neighbourhoods of Blantyre remains speculative.

Future priorities include investigation of the prevalence of undiagnosed TB in neighbourhoods with low CNRs but high poverty and poor access to care indicators, where TB may be underdiagnosed: if so, then the prevalence of undiagnosed infectious TB may in fact be very high in these areas. Community-level TB interventions including active case finding have tended to focus on high-density informal residential areas, guided by high CNRs and prevalence survey data that show urban settings to be high risk for TB [26]. Even within densely populated urban settings such as Blantyre, there may be substantial heterogeneity in TB prevalence and risk factors for transmission, meaning that the potential effects of community-wide interventions may become diluted. We note that across 3 years studied, there were consistent patterns of high TB case notifications arising from the same set of CHW catchment areas. It is possible that case-finding interventions directed towards areas with the greatest need (both in terms of risk factors for TB and in likely under-diagnosis) could have greater and quicker impact on controlling TB than broader population-based strategies. However, further epidemiological and modelling studies, as well as trials, are required.

## Conclusions

In summary, we found evidence from statistical analysis of enhanced city-wide surveillance data that the poorest neighbourhoods of Blantyre, and those farthest from TB diagnosis and treatment clinics, had substantially lower relative TB CNRs. This remained true after adjusting for important epidemiological risk factors for TB, implying inequity in access to diagnosis and care. The innovative georeferenced surveillance system described here could be implemented widely in low-resource settings and potentially provide high-quality, real-time epidemiological and microbiological data to direct TB control efforts. In particular, the impact on TB burden of facilitated access to diagnosis and spatially directed active TB case-finding interventions should be examined further.

## Additional files

Additional file 1: **Figure S1.** Characteristics of Health Surveillance Assistant catchment areas, Blantyre, Malawi. A) Total population; B) population density per km^2^; C) Mean number of people per household; D) Mean proportion of population living in poverty; E) Cartesian distance from catchment area centroid to nearest TB registration centre; F) Ratio of male-to-female adults G) Proportion of population aged 15 years or older H) Sputum smear positive to negative ratio, on sample from routine clinic. Poverty estimates from Worldpop (www.worldpop.org.uk). Boundaries are Health Surveillance Assistant catchment areas. Black triangles are TB registration clinic. White areas in centre of maps are mountainous areas or business districts with few residents that were not enumerated in the census. The black triangle in the far north of maps is a health centre with a TB registration clinic located outside of Blantyre District that may be used by Blantyre residents; to increase accuracy of case notification rates, we captured TB registrations at this clinic. (PDF 933 kb)

Additional file 2: **Figure S2.** Nearest neighbour structure of Blantyre Health Surveillance Assistant catchment areas. Blue lines indicate the nearest *k* neighbours to each 315 Health Surveillance Assistant catchment area, calculated as the Cartesian distance between pairs of Health Surveillance Assistant catchment area centroids. (PDF 37 kb)

Additional file 3: **Figure S3.** Sensitivity analysis. X-axes are relative rates per unit increase in variable. Model k0 is model without spatial autocorrelation prior. Models k1 to k6 are models with *k* = 1 to 6 nearest neighbour matrix spatial autocorrelation priors. *k* nearest neighbours calculated from Cartesian distances between pairs of Health Surveillance Assistant catchment area centroids (Additional file 2: Figure S2). WAIC: Widely applicable information criteria based on the posterior distribution of each model. (PDF 14 kb)

Additional file 4: **Figure S4.** Predicted and observed TB cases in 315 Blantyre Health Surveillance Assistant catchment areas. A: All TB cases. B: Microbiologically confirmed TB cases. X-axes are Health Surveillance catchment areas, ordered in decreasing frequency by number of TB cases registered through enhanced TB surveillance. Red points are observed numbers of cases, blue points are predicted values, estimated by the Bayesian spatial regression models. Transparency of blue fitted points are inversely proportional to absolute distance from observed value. Predicted numbers of TB cases estimated by fitting a Bayesian spatial regression model with Poisson response, a k = 6 nearest-neighbours conditional spatial autocorrelation structure, with linear terms fitted for health surveillance assistant catchment area log_10_ total population, log_10_ population density, adult M:F ratio, mean number of people per household, log_10_ Cartesian distance from geographical centroid to the nearest TB clinic, proportion of population aged 15 years or older, mean proportion living on less than US $2 per day, sputum smear positive to negative ratio, offset term for log_10_ HSA population size, and with weakly informative prior on the population-level effects intercept (Gaussian: mean = 0, sd = 10), and predictor intercept (Gaussian, mean = 0, sd = 10). (PDF 109 kb)

## Abbreviations

CHW: Community health worker
CNR: Case notification rate
ePAL: Electronic patient locator
HIV: Human immunodeficiency virus
MCMC: Markov chain Monte Carlo
NUTS: Non-U-turn sampler
TB: Tuberculosis
WAIC: Widely applicable information criteria
WHO: World Health Organization
