# Supplementary figures and images for: Disparities in access to diagnosis and care in Blantyre, Malawi, identified through enhanced tuberculosis surveillance and spatial analysis
Source: BMC Med. 2019 Jan 29;17:21. doi: 10.1186/s12916-019-1260-6 (PMC6350280; doi:10.1186/s12916-019-1260-6)

**A**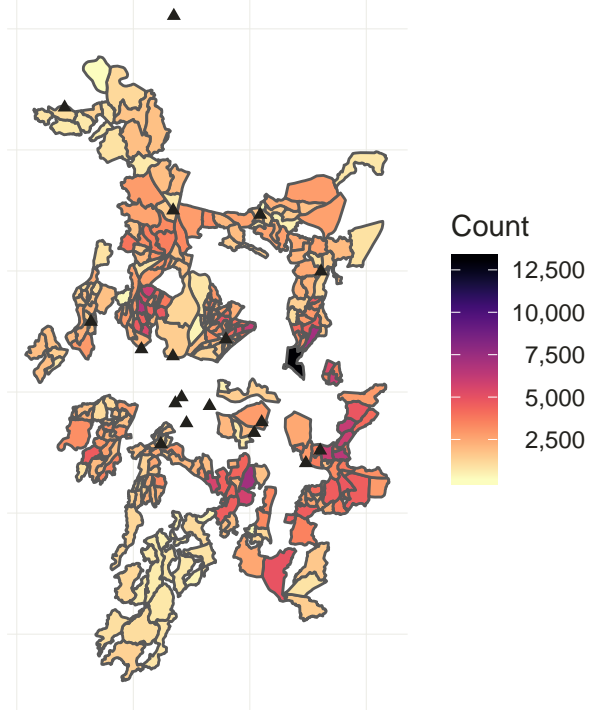**B**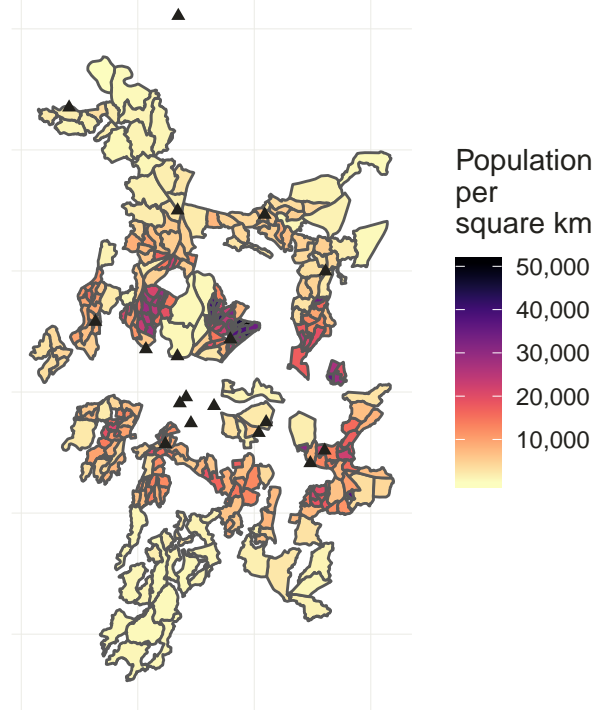**C**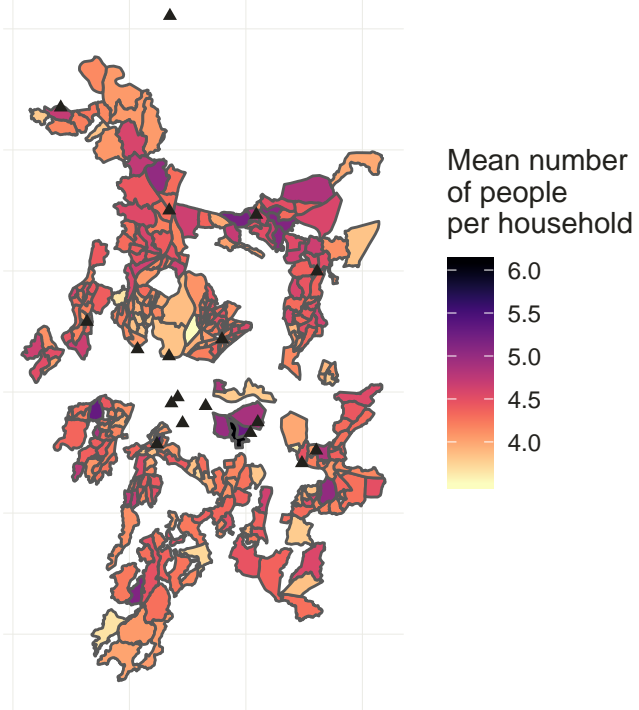**D**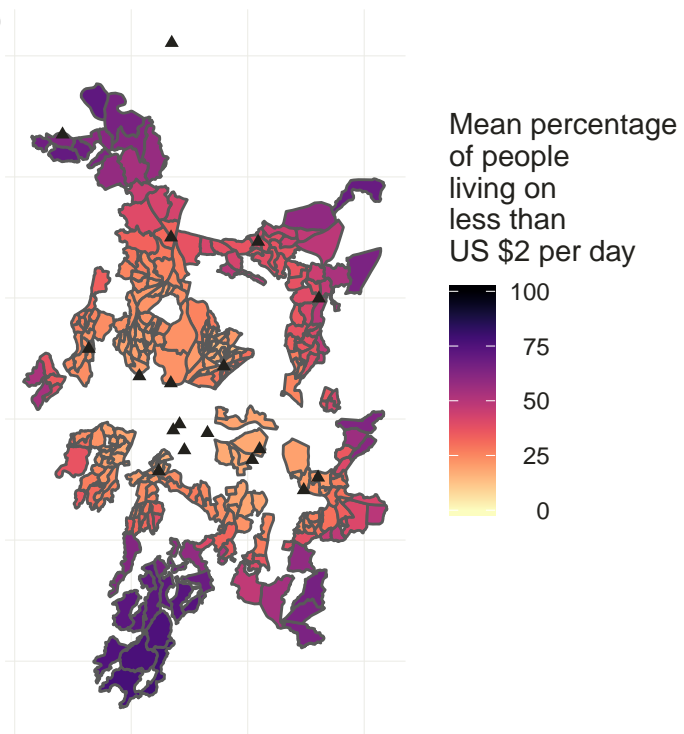**E**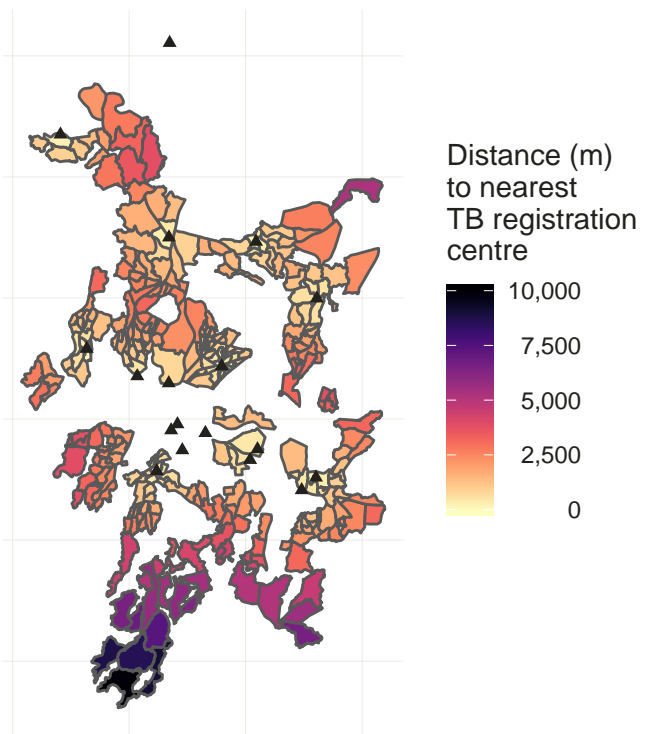**F**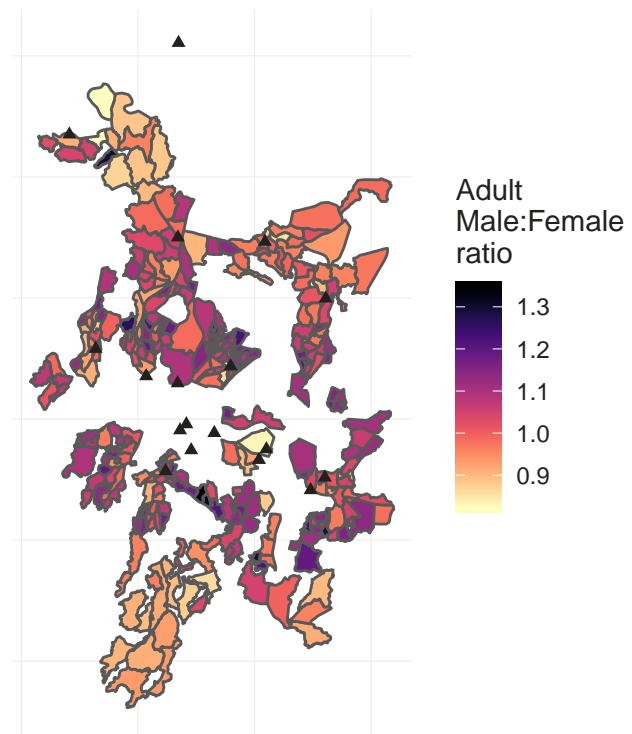**G**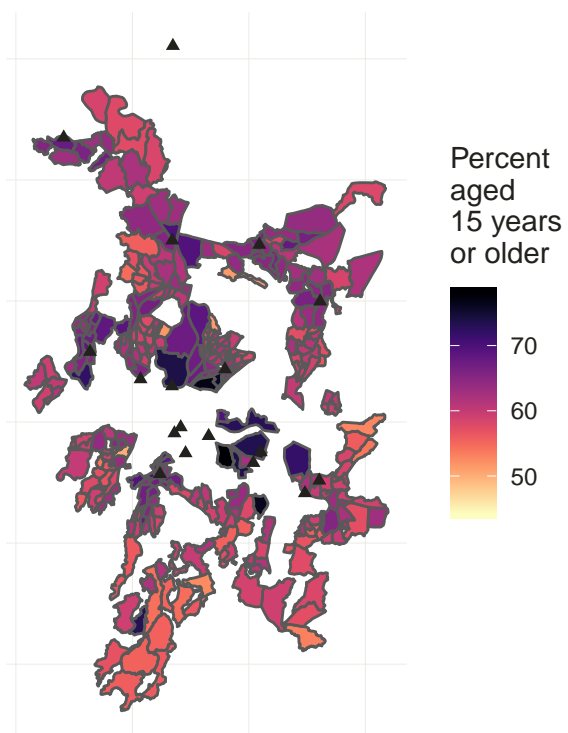**H**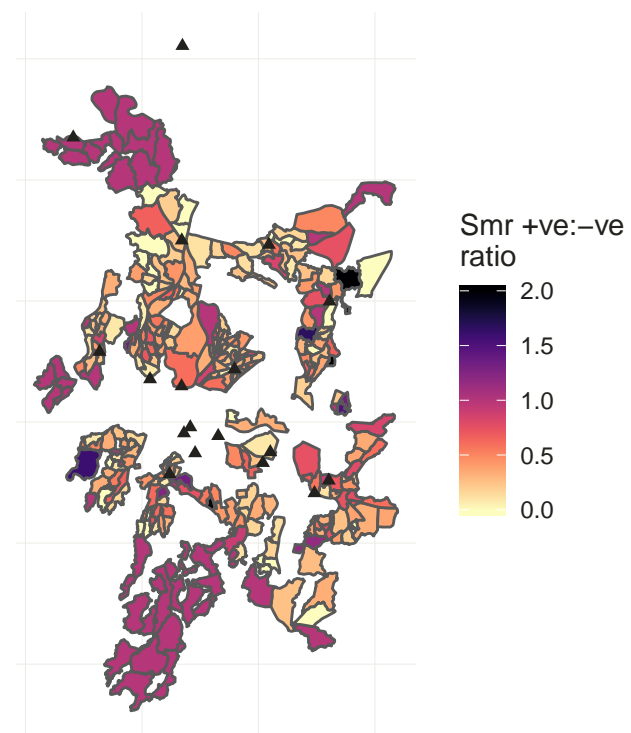

Supplement: Supplementary file 1 — Figure S1. Characteristics of Health Surveillance Assistant catchment areas, Blantyre, Malawi. A) Total population; B) population density per km2; C) Mean number of people per household; D) Mean proportion of population living in poverty; E) Cartesian distance from catchment area centroid to nearest TB registration centre; F) Ratio of male-to-female adults G) Proportion of population aged 15 years or older H) Sputum smear positive to negative ratio, on sample from routine clinic. Poverty estimates from Worldpop (www.worldpop.org.uk). Boundaries are Health Surveillance Assistant catchment areas. Black triangles are TB registration clinic. White areas in centre of maps are mountainous areas or business districts with few residents that were not enumerated in the census. The black triangle in the far north of maps is a health centre with a TB registration clinic located outside of Blantyre District that may be used by Blantyre residents; to increase accuracy of case notification rates, we captured TB registrations at this clinic. (PDF 933 kb) [file 12916_2019_1260_MOESM1_ESM.pdf]

**k=1**

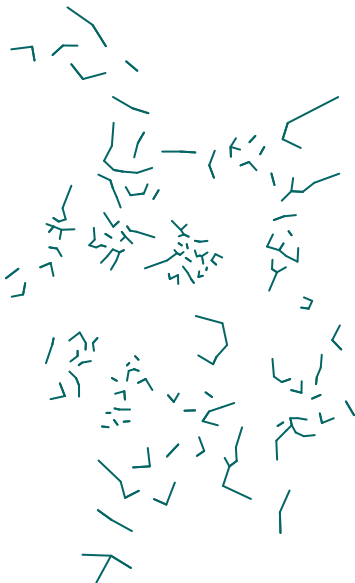

**k=2**

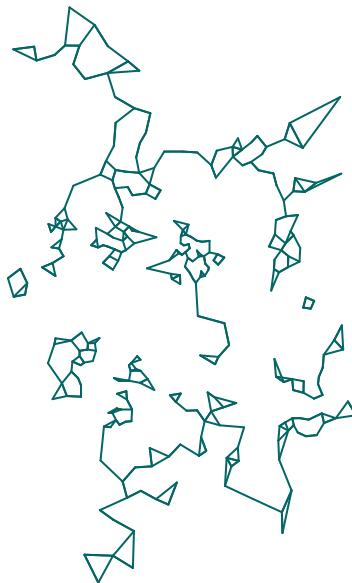

**k=3**

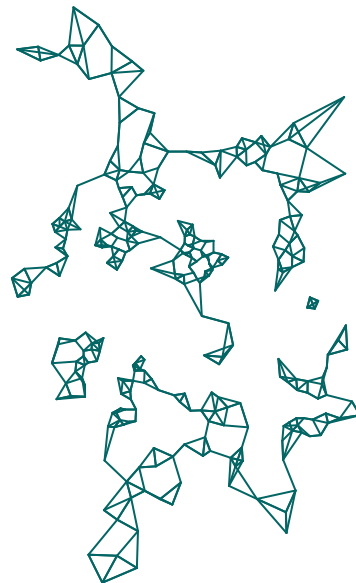

**k=4**

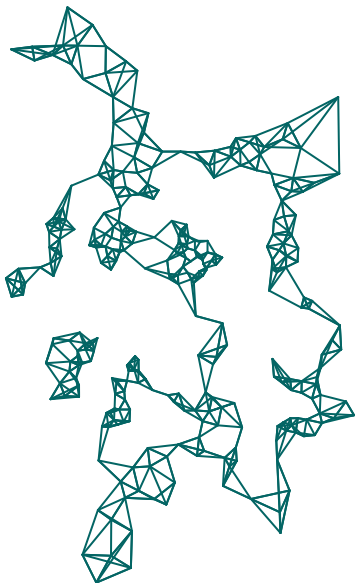

**k=5**

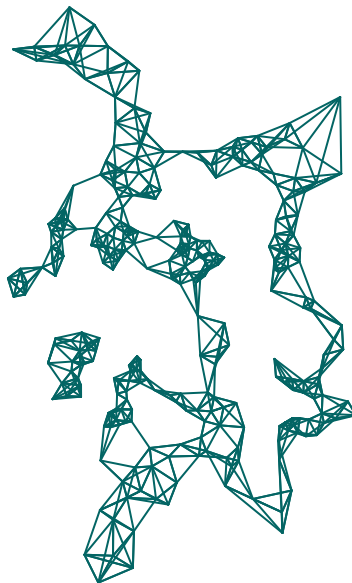

**k=6**

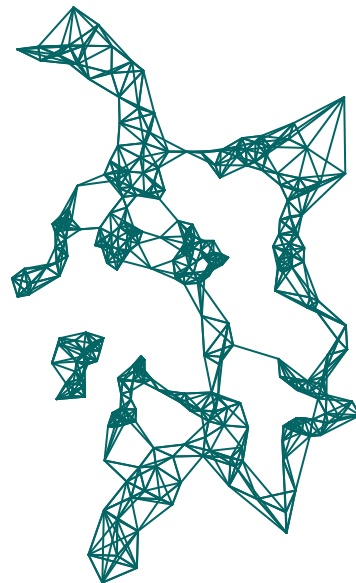

Supplement: Supplementary file 2 — Figure S2. Nearest neighbour structure of Blantyre Health Surveillance Assistant catchment areas. Blue lines indicate the nearest k neighbours to each 315 Health Surveillance Assistant catchment area, calculated as the Cartesian distance between pairs of Health Surveillance Assistant catchment area centroids. (PDF 37 kb) [file 12916_2019_1260_MOESM2_ESM.pdf]

# A

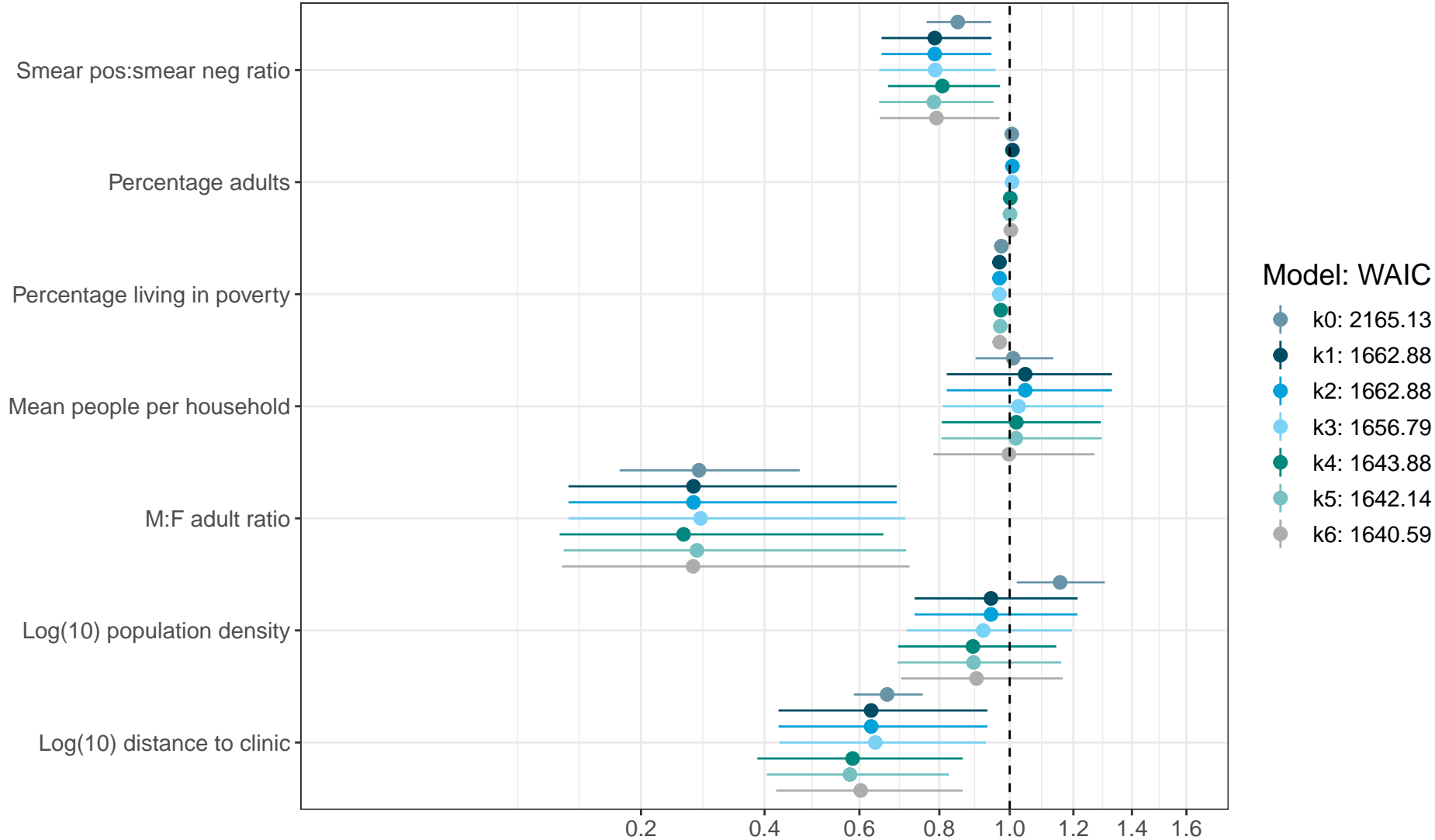

**B**

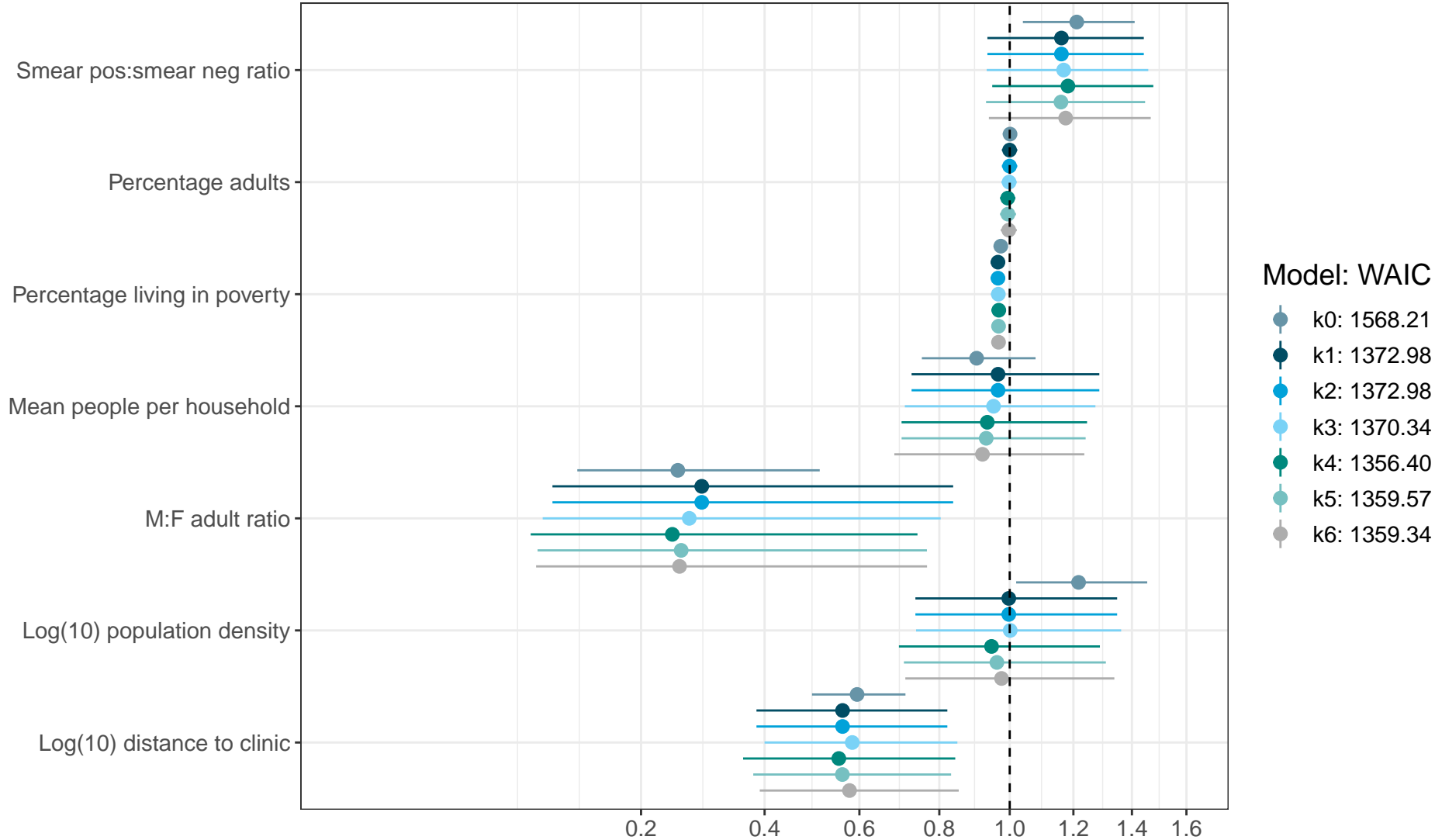

Supplement: Supplementary file 3 — Figure S3. Sensitivity analysis. X-axes are relative rates per unit increase in variable. Model k0 is model without spatial autocorrelation prior. Models k1 to k6 are models with k = 1 to 6 nearest neighbour matrix spatial autocorrelation priors. k nearest neighbours calculated from Cartesian distances between pairs of Health Surveillance Assistant catchment area centroids (Additional file 2: Figure S2). WAIC: Widely applicable information criteria based on the posterior distribution of each model. (PDF 14 kb) [file 12916_2019_1260_MOESM3_ESM.pdf]

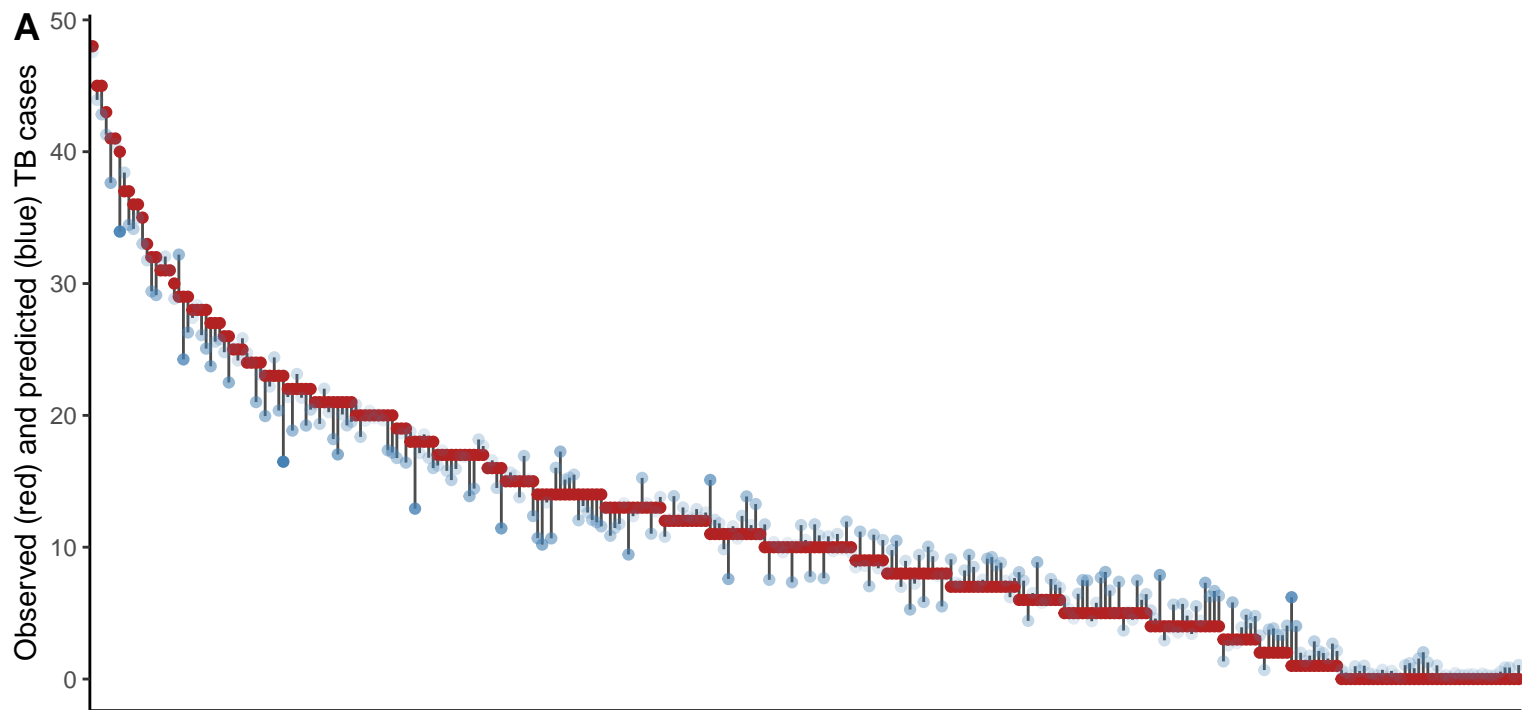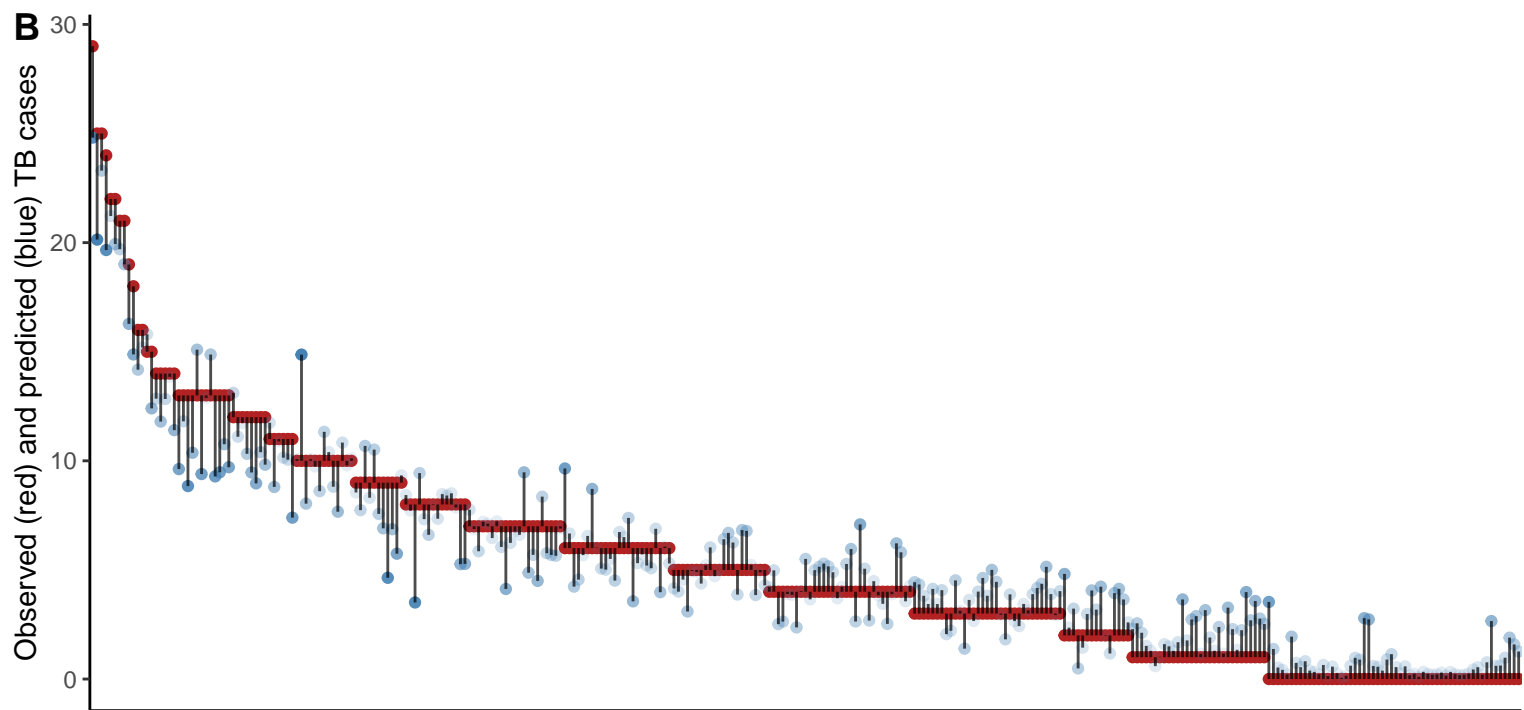

Supplement: Supplementary file 4 — Figure S4. Predicted and observed TB cases in 315 Blantyre Health Surveillance Assistant catchment areas. A: All TB cases. B: Microbiologically confirmed TB cases. X-axes are Health Surveillance catchment areas, ordered in decreasing frequency by number of TB cases registered through enhanced TB surveillance. Red points are observed numbers of cases, blue points are predicted values, estimated by the Bayesian spatial regression models. Transparency of blue fitted points are inversely proportional to absolute distance from observed value. Predicted numbers of TB cases estimated by fitting a Bayesian spatial regression model with Poisson response, a k = 6 nearest-neighbours conditional spatial autocorrelation structure, with linear terms fitted for health surveillance assistant catchment area log10 total population, log10 population density, adult M:F ratio, mean number of people per household, log10 Cartesian distance from geographical centroid to the nearest TB clinic, proportion of population aged 15 years or older, mean proportion living on less than US $2 per day, sputum smear positive to negative ratio, offset term for log10 HSA population size, and with weakly informative prior on the population-level effects intercept (Gaussian: mean = 0, sd = 10), and predictor intercept (Gaussian, mean = 0, sd = 10). (PDF 109 kb) [file 12916_2019_1260_MOESM4_ESM.pdf]
